# Supplementary material for: Confidence drives a neural confirmation bias
Source: Nat Commun. 2020 May 26;11:2634. doi: 10.1038/s41467-020-16278-6 (PMC7250867; doi:10.1038/s41467-020-16278-6)
Supplement: Supplementary file 1 — Supplementary Information [file 41467_2020_16278_MOESM1_ESM.pdf]

Confidence drives a neural confirmation bias

Supplementary Information

Rollwage et al.

Supplementary Note 1: Adaptive use of post-decision evidence

In the main text we focus on an influence of confidence on post-decision evidence processing and changes of mind, with the assumption that disregarding new information (i.e. showing a confirmation bias) is maladaptive. Here, we report additional analysis showing that using post-decision evidence is indeed adaptive in our task.

First we established that subjects improved their choice accuracy from the initial to the final decision ($t(27)= 7.45$, $p<10^{-7}$, see Supplementary Figure 1A), showing that the new evidence was beneficial. Specifically, this increase in accuracy was due to participants changing their mind more often when they were initially incorrect compared to when they were correct ($t(27)=13.39$, $p<10^{-12}$, see Supplementary Figure 1C), indicating that participants changed their mind to reverse mistakes. This improvement in accuracy was accompanied by a decrease in reaction times of the final decision compared to those of initial decisions ($t(27)= 3.21$, $p=.003$, see Supplementary Figure 1B). We also examined participants' final confidence ratings (Supplementary Figure 1D, a confidence rating below 50% indicates that participants changed their mind) as a function of post-decision evidence strength, showing that participants reduced their confidence in initially erroneous decisions ($t(27)= 7.38$, $p<10^{-7}$) and that this effect was most pronounced when post-decision evidence was strong ($t(27)= 3.03$, $p=.005$).

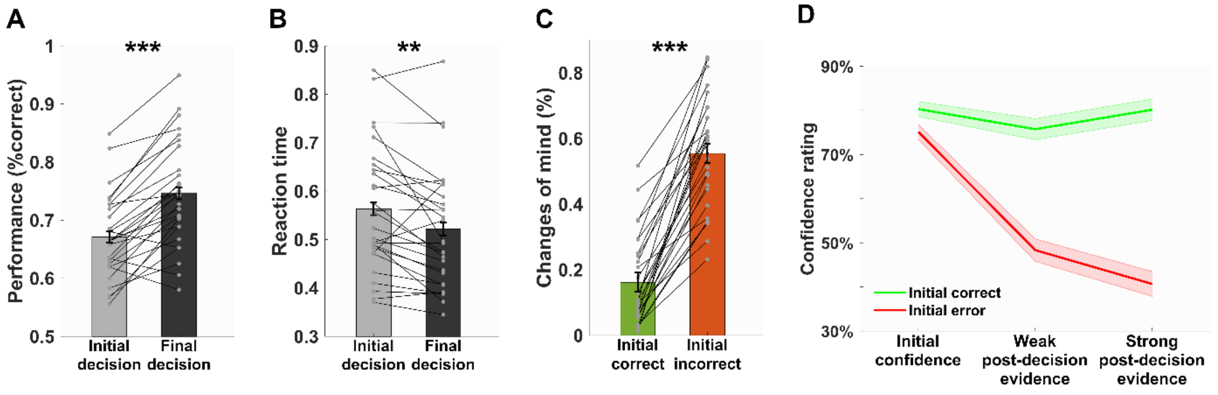

Supplementary Figure 1 *Influence of post-decision evidence on the final decision (behavioural study 1: n=28 participants).* ***A*** *Performance for the initial and final decision.* ***B*** *Reaction times for the initial and final decision.* ***C*** *Proportion of changes of mind after initially correct and incorrect choices.* ***A-C*** *Data are presented as mean values ± SEM; grey dots represent*

individual participant data. ***D*** *Participants decreased their confidence in initial errors as a function of post-decision evidence strength (confidence levels below 50% indicates changes of mind). Lines represent group average and shaded area represents ± SEM. Paired t-test (two-tailed):* ***A)*******$p=10^{-7}$,* ***B)******$p=.003$,* ***C)*******$p=10^{-12}$*

Supplementary Note 2: Model comparison and simulations of drift-diffusion models

In total, we compared 10 different drift-diffusion models (Supplementary Figure 2A, see Methods for further description of the models) that differed in whether the starting point and/or drift-rate were affected by confidence (models 2-4), initial decision (model 5-7) and their interaction (model 8-10). A full model (model 10) with dependencies of starting point and drift-rate on all factors (confidence, initial decision and the interaction) was the preferred model based on the Deviance Information Criterion (DIC) score, and provided a good fit to the data (Figure 2B and C). Critically, the model fits indicated that evidence accumulation started closer to the initially chosen decision bound when people were highly confident ($p < 10^{-20}$; see Figure 2D). Additionally, high confidence led to a bias in evidence accumulation in favour of the initial choice, consistent with a selective accumulation of evidence in support of an initial decision ($p < 10^{-20}$; see Figure 2D).

The lower DIC of models that included dependencies of starting point and drift-rate on the interaction between confidence × initial decision (ΔDIC=151.6 relative to the best model without an interaction term) indicated that a subset of data features could only be captured by modelling this interaction term. Indeed, the winning model closely accounted for observed interactions between confidence and initial decision in both accuracy and response times (see Figure 2B&C). To visualize how the model fits deviated from the data when this interaction term was omitted, we also simulated model predictions from the fits of other model families (Supplementary Figure 2B and C).

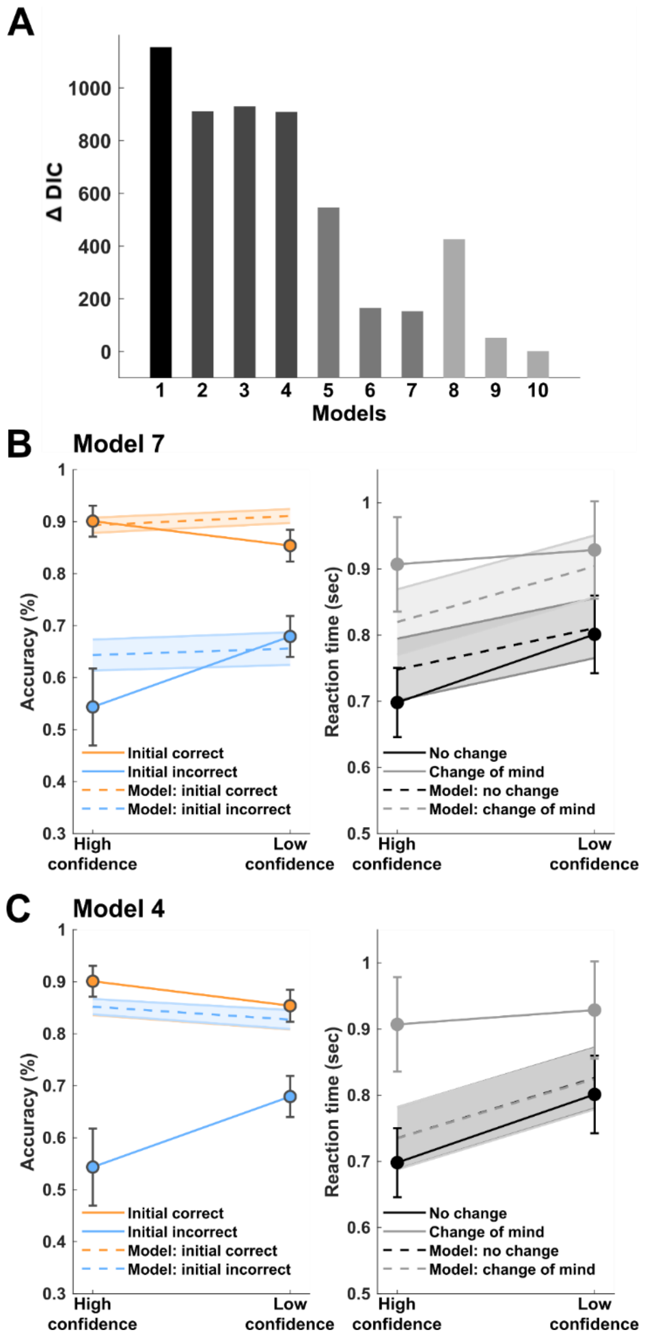

Supplementary Figure 2 *Comparison and simulation of alternative drift-diffusion models (n=23 participants).* ***A*** *Model comparison of the 10 alternative drift-diffusion models. Models were compared based on the DIC where lower values indicate better fit. We present ΔDIC representing the difference of each model's DIC against the best fitting model in the set (the best fitting model 10 has a ΔDIC of zero). Model 1 is a baseline model with no dependencies of drift-rate or starting point. Models 2-4 represent a model family in which the drift-rate and/or starting point are affected by initial confidence. Models 5-7 represent a model family in which the drift-rate and/or starting point are affected by the initial decision (i.e. a simple confirmation bias). Models 8-10 represent a model family in which the drift-rate and/or starting point are affected by initial confidence, initial decision and their interaction (i.e. a confirmation bias boosted or attenuated by confidence).* ***B-C*** *Simulations of alternative models. Model simulations are shown as dotted lines and behavioural data as solid lines. Data are presented as mean values +/- 95% confidence interval.*

Supplementary Note 3: Quantifying the contribution of confidence effects on starting point and drift-rate

The comparison of drift-diffusion models in Supplementary Note 2 (previous section) revealed that confidence, initial decision and their interaction showed influences on both the starting point and the drift-rate. We next quantified the relative contribution of both effects to the observed data patterns. To this end, we started with the best-fitting model (model 10) and eliminated either the dependencies of the starting point or the drift-rate. Eliminating starting point dependencies reduced the model fit only slightly (ΔDIC=48.1), whereas elimination of drift-rate dependencies reduced the model fit severely (ΔDIC=425.6). Accordingly, when comparing these two models directly (which are matched for complexity), the model with drift-rate dependencies explained the data better than the model with starting point dependencies (ΔDIC=377.5). This indicates that a confirmation bias, and a boost of confirmation bias through confidence, is better explained by selective accumulation of choice consistent information rather than a shift in starting point.

Supplementary Note 4: Validating neural metrics of evidence accumulation during the pre-decision period

We trained a machine-learning classifier to predict participants' initial choices (left versus right) based on whole-brain activity (normalized amplitude of all MEG channels). The probabilistic prediction of this classifier within each 10ms timebin within a trial provides a neural metric of internal evidence for a left versus right decision. We reasoned that changes in this neural representation over time within a trial in response to presented evidence (i.e. moving dots) provides an indicator of how evidence is accumulated. We summarized this build-up of internal evidence within a trial by fitting a linear regression to all time points, giving us a summary statistic for the rate of change of the representation of internal evidence (slope of the linear regression) and its starting point (intercept of the linear regression) on every trial.

In the main text we focus on the reapplication of those classifiers to the post-decision phase as our measure of post-decision evidence integration. Here we report additional analysis to validate this approach as a metric of evidence accumulation in the pre-decision phase.

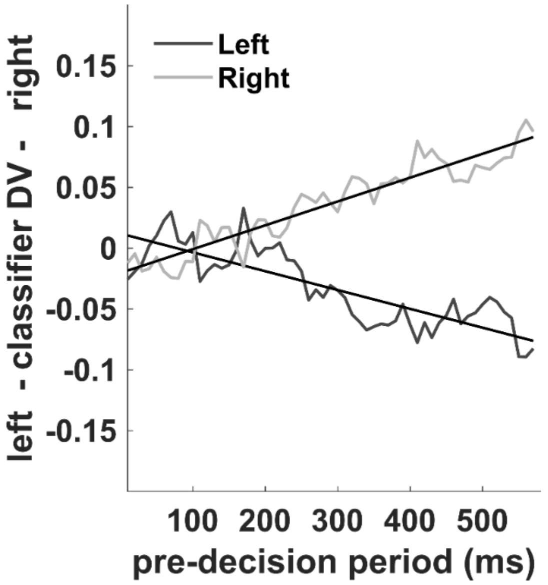

Supplementary Figure 3 *Group average of the left/right classifier prediction in response to pre-decision evidence (n=25 participants). The light gray line shows the change in neural representation when rightward motion is presented and the black line shows the change in neural representation when lefttward motion is presented. The regression lines are the fit to the group-averaged data for visualisation purposes.*

First, a measure of evidence accumulation (slope) should be responsive to the veridical motion direction presented which was the case for the pre-decision phase (β=. 16, $p<10^{-13}$, see Supplementary Figure 3). Second, an internal decision variable should be related to characteristic features of the observer's decision, over and above this responsiveness to external features of the stimulus. Specifically, stronger internal evidence accumulation should be related to a higher likelihood of making a correct decision[1], faster response times[2] and higher confidence[3]. To evaluate these predictions we computed the unsigned change in classifier prediction to provide a metric of the strength of evidence accumulation irrespective of whether this was in favour of a leftward or rightward decision.

We then entered both the slope and intercept of neural evidence accumulation on each trial in a hierarchical multiple regression model to predict a) choice accuracy, b) reaction times and c) confidence in the pre-decision phase. Steeper slopes predicted faster reaction times (β=-0.001, p<.01, see Supplementary Figure 4A), a higher likelihood of a correct decision (β=0.28, $p<10^{-10}$, see Supplementary Figure 4B) and higher confidence (β=0.15, p<.0001, see Supplementary Figure 4C). We also asked whether slope metrics predicted behaviour independently of maximum classifier prediction achieved within a trial (i.e. at the timepoint of highest decodability). When controlling for the classifier prediction at the timepoint of highest decodability, the slope remained a significant predictor of reaction time (β=-0.005, p=.048), accuracy (β=0.1, p=.001) and confidence (β=0.05, p=.027). While the intercept had no effect on reaction times or accuracy (all p-values >.05), there was a significant effect on confidence (β=-0.08, p=.01), consistent with pre-decisional influences on decisions[4] and confidence ratings[5] even in the absence of a serial dependency in task structure.

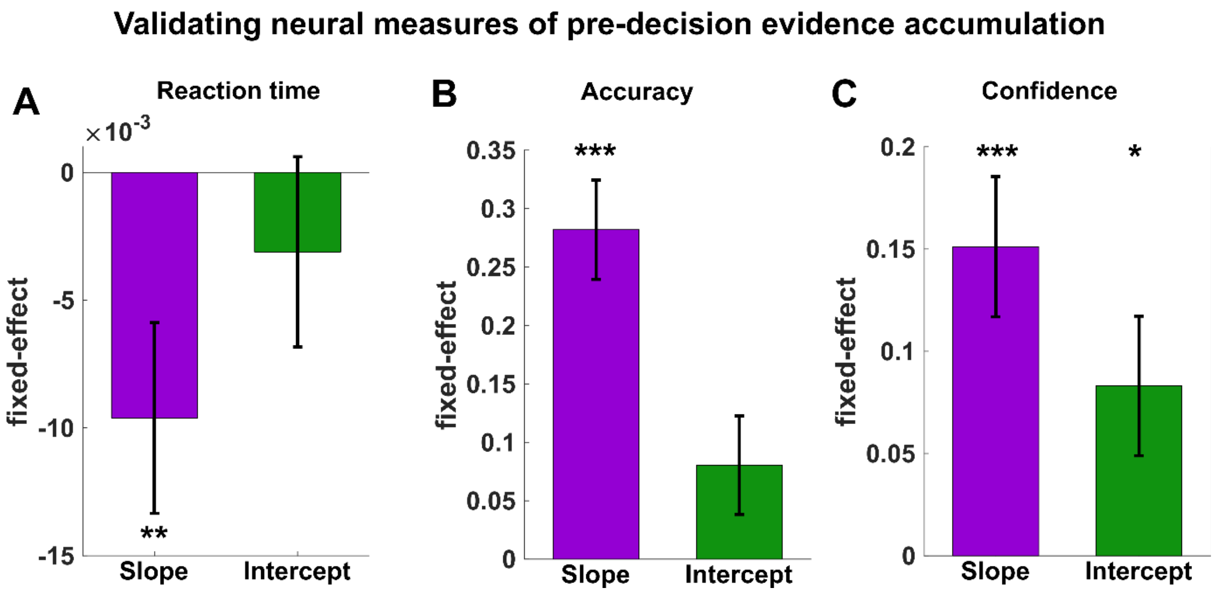

Supplementary Figure 4 *Validation of neural metrics of pre-decision evidence accumulation (n=25 participants).* ***A-C*** *Neural measures of the slope and starting point (intercept) of evidence accumulation extracted from the pre-decision phase were entered as simultaneous predictors of* ***A****) reaction times* ***B****) accuracy and* ***C****) confidence of the initial decision. Fixed effects from a hierarchical regression model are presented ± SEM. Hierarchical regression (two-tailed):* ***A)*** ***p=.009;* ***B)*** ****$p=10^{-10}$;* ***C)*** **p=.01, ***p=.0001*

Supplementary Note 5: Effects of confidence on changes of mind – behavioural analysis

Here we report additional behavioural and MEG analyses that corroborate a distinct influence of confidence on changes of mind.

First, when focussing on subjects for whom the positive evidence manipulation led to a boost in confidence (blue dots in Figure 1D) we observed a reduction in changes of mind ($t(20)=3.51$, $p=.002$) which was fully mediated by the confidence change ($a \times b$; $\beta= -4.84$, $p<10^{-6}$; Supplementary Figure 5B; this effect was replicated in behavioural study 2: $\beta=-3.39$, $p<0.0001$; and MEG study 3: $\beta=-10.7$, $p<.0001$). Second, we used trial-by-trial confidence ratings to predict trial-by-trial changes of mind while statistically controlling for accuracy and reaction times. We implemented a hierarchical logistic regression model with changes of mind as dependent variable (1= change of mind, 0= no change of mind) and entered the following predictors: confidence in the initial decision, accuracy of the initial decision (1= correct, 0 = incorrect), reaction time of the initial decision, post-decision evidence (PDE) strength (1 = weak, 2 = strong PDE) and the accuracy × PDE strength interaction. The rationale for entering the final interaction term was as follows: since PDE was always helpful (i.e. the predominant movement direction was the same as in the pre-decision phase) we expected subjects to change their minds less often as a function of PDE strength if this evidence was in line with their initial decision (i.e. their initial choice was accurate).

Consistent with the findings reported in the main text, higher confidence in an initial decision reduced the probability participants would change their minds in the face of new evidence (behavioural study 1: $\beta = -2.0$, $p < 0.0001$, see Supplementary Figure 5 C), while controlling for choice accuracy and reaction times. This effect was replicated across all studies (behavioural study 2: $\beta = -1.57$, $p = 0.0002$, MEG study 3: $\beta = -0.56$, $p < 10^{-6}$), lending support

to its robustness and indicating that a subjective feeling of confidence makes a distinct contribution to the propensity to change one's mind.

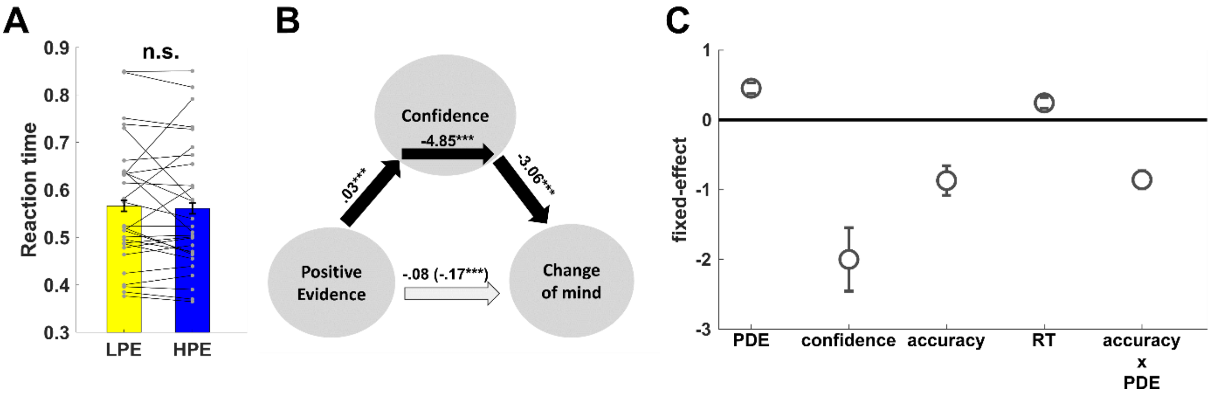

Supplementary Figure 5 A *Reaction times of the initial decision in the high and low positive evidence conditions, showing that manipulating positive evidence did not systematically alter reaction times (n=28 participants). Data are presented as mean values ± SEM; grey dots represent individual participant data.* ***B*** *Multi-level mediation analysis revealed that changes in confidence mediated the impact of positive evidence on changes of mind (n=21 participants).* ***C*** *Results of a hierarchical logistic regression predicting changes of mind, showing that confidence predicts changes of mind while statistically controlling for performance variables including accuracy and reaction time. Group-level fixed-effects ± SEM are presented (n=28 participants). Data are from behavioural study 1. LPE = low positive evidence condition, HPE = high positive evidence condition, PDE = post-decision evidence strength, RT = Reaction time. Multi-level mediation (two-tailed): ***p<.001.*

Supplementary Note 6: Effects of confidence on changes of mind – neural analysis

In the previous section we used regression modelling to isolate a distinct influence of confidence on changes of mind. We also ensured that our psychophysical manipulation of positive evidence (see main text) led to changes in confidence while keeping task performance and reaction times constant. Here we harnessed our MEG data to further dissociate those factors and ask how neural signatures of confidence and evidence strength determine later changes of mind.

To this end, we exploited a similar machine-learning classification approach to that described in the main text. Here we focussed on the pre-decision phase (from pre-decision stimulus onset to the presentation of choice options of the initial decision, an 850 ms time window), as we were interested in which features of the initial decision predict whether people later change their mind. We trained two separate classification algorithms, one to predict participants' confidence ratings (high or low) and the other to predict participants' decisions (left or right). We identified time points within the pre-decision window with the highest decodability (separately determined for each classifier and each participant) for use in subsequent regression analyses. As explained in the main text, our classification algorithm fits a hyperplane that best separates the two classes of trials and the distance of each trial to this boundary provides a graded metric of the evidence supporting the classification, which we interpret here as the strength of a neural representation of either the decision or confidence level[6,7]. To obtain an absolute metric of evidence strength from the decision classifier, we further computed the unsigned value of the classifier prediction for leftward or rightward decisions on each trial. We could then ask which of these two neural representations (decision or confidence) best predict whether or not a person will change her mind.

First, we established that we were able to decode both representations above chance. This was the case (see Supplementary Figure 6A), with decisions being decodable earlier in time than confidence and showing an earlier peak of highest decodability (Wilcoxon signed rank test, $p=0.051$). A time-lag analysis of the decodability time series (i.e. shifting both time-series against each other to identify the delay at which both time-series show the strongest correspondence) also confirmed that decisions were decodable earlier in time than confidence ratings with a lag of around 150ms (see Supplementary Figure 6B).

When investigating the topography of features (i.e. channel activity) that contributed to the decoding of confidence and decisions, we revealed that the two classifiers also picked up distinct brain activity patterns (see Supplementary Figure 6C&D). The classification algorithm that predicted left versus right decisions mainly utilized activity in centro-parietal regions to predict participants upcoming decisions (see Supplementary Figure 6D). In contrast, the confidence classifier utilized patterns of central, frontal and occipital sensors (see Supplementary Figure 6C). The occipital contribution to confidence classification was observed late within a trial, after the stimuli had disappeared from the screen, indicating that this contribution may reflect a top-down effect on occipital regions.

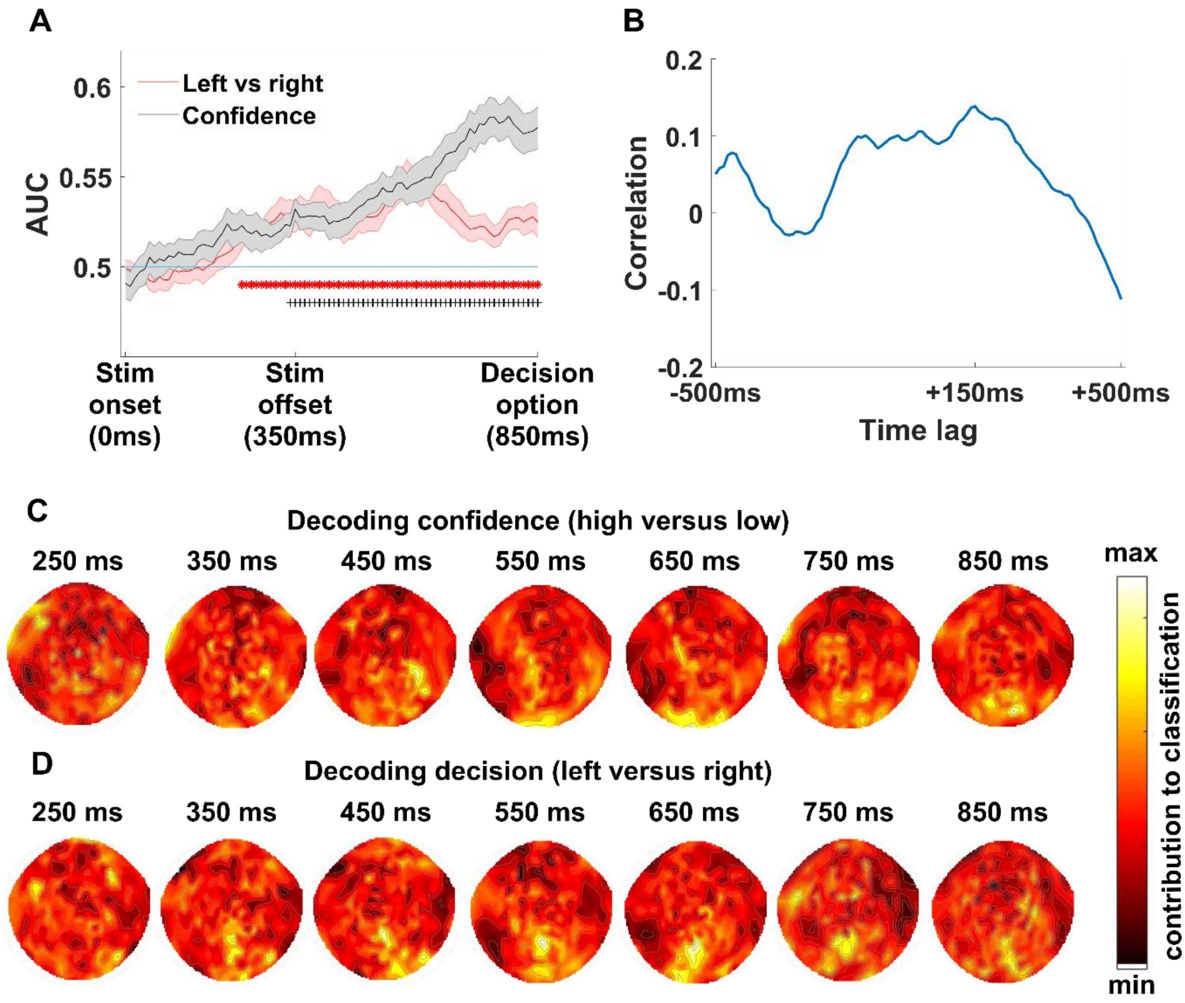

Supplementary Figure 6 *MEG analysis indicating distinct neural representations of confidence and decision (n= 25 participants).* ***A*** *We trained two separate support-vector machine learning classification algorithms on MEG activity during the pre-decision phase of the trial to predict participants' decisions (left or right, red line) and confidence ratings (low or high, black line). Both decisions and confidence were decodable above chance level (based on permutation testing), indicated by asterisks, although decisions were decodable earlier in time in comparison to confidence. Data are presented as group mean values +/- SEM.* ***B*** *Time-lag analysis between the decoding accuracy time course for decisions and confidence. This analysis shifts the decision and confidence decoding timecourses against each other across multiple time-lags to determine the lag at which the overlap (i.e. correlation) between both timecourses is the highest (positive values indicate that confidence decoding occurs later in time). The confidence decoding time course follows the decision decoding time course with a peak lag of 150 ms.* ***C&D*** *Contributions of sensors to decoding* ***C)*** *high versus low confidence ratings and* ***D)*** *left versus right decisions. To explore which brain areas carried information about each classification type, we trained support-vector machine classifiers for each participant at different time points of the trial (250-850 ms), repeating each analysis 2500 times using subsets of 30 sensors randomly selected on each iteration. The contribution of sensor* s *was defined as the mean of all prediction accuracies achieved using an ensemble of 30 sensors that included* s*. Group averages of the relative contribution for each sensor are presented.*

We next asked how the strengths of these neural representations (decision and confidence) are related to changes of mind. The unsigned neural representation strength of the decision classifier did not predict whether people changed their mind later on ($\beta=-.05$, $p=.3$, see Supplementary Figure 7A). In contrast, a neural representation of confidence was highly predictive of changes of mind ($\beta=-.49$, $p<10^{-68}$, see Supplementary Figure 7A), with changes of mind being less likely when the MEG activity profile was indicative of high confidence in a decision. We also considered whether the slope of internal evidence accumulation carries additional information predictive of later changes of mind (see Supplementary Note 4). Interestingly, slopes fitted to the change in neural representation of the decision classifier were predictive of later changes of mind ($\beta=-.12$, $p<10^{-5}$, see Supplementary Figure 7B). The neural representation of confidence remained a significant predictor when including decision classifier slope as a covariate ($\beta=-.49$, $p<10^{-68}$, see Supplementary Figure 7B) and confidence was a significantly stronger predictor than decision classifier slope ($t(17098)=9.53$, $p<10^{-20}$). These results indicate that neural activity patterns covarying with confidence, over and above activity predictive of first-order decision accuracy, are key predictors of whether or not a person will change her mind.

In a further analysis step, we also investigated whether a neural representation of confidence was able to predict subsequent changes of mind over and above behavioural confidence ratings. To this end, we constructed a similar hierarchical regression model but now separated into trials in which people reported high or low confidence. Notably, variations in a neural representation of confidence remained predictive of whether or not participants changed their mind, despite explicit ratings being equated (low confidence ratings: $\beta=-.22$, $p<.0001$, high confidence ratings: $\beta=-.28$, $p<10^{-6}$). It is of course possible that a confidence scale with greater

granularity would capture greater variance in changes of mind than achievable from a binary rating.

A positive evidence manipulation was employed in the MEG study, leading to higher confidence in the high positive evidence condition ($t(24)=3.27$, $p=0.003$), which in turn reduced changes of mind ($t(15)=-3.47$, $p=0.003$) for those subjects showing the expected effect on confidence. Using a multi-level mediation analysis, we asked whether this effect was driven by inducing stronger confidence-related neural activity or by altering internal evidence strength. The effect of the positive evidence manipulation on changes of mind (c: $\beta=-0.15$, $p<0.05$) was completely mediated by a change in neural representation of confidence (see Supplementary Figure 7C), leading to a significant indirect effect (a×b: $\beta=-3.36$, $p=.002$). In contrast, no mediation was observed when entering the strength of neural representation from the decision classifier as a mediating variable, with no significant indirect effect (a×b: $\beta=-.07$, $p=0.4$, see Supplementary Figure 7D). A direct effect of positive evidence on changes of mind also remained unchanged after controlling for the strength of neural representation from the decision classifier (c': $\beta=-0.15$, $p<0.05$). Finally, we established that a neural representation of confidence also predicted changes of mind within each positive evidence condition in separate hierarchical regression models. In both high and low positive evidence conditions, a neural representation of confidence predicted fewer changes of mind (HPE: $\beta=-.5$, $p<10^{-35}$; LPE: $\beta=-.47$, $p<10^{-33}$).

Together, our MEG and behavioural results indicate confidence as a distinct factor predicting changes of mind – one that cannot be explained by appeal to variation in internal evidence strength. More broadly, these analyses support a model in which confidence acts as top-down controller of subsequent information processing[8].

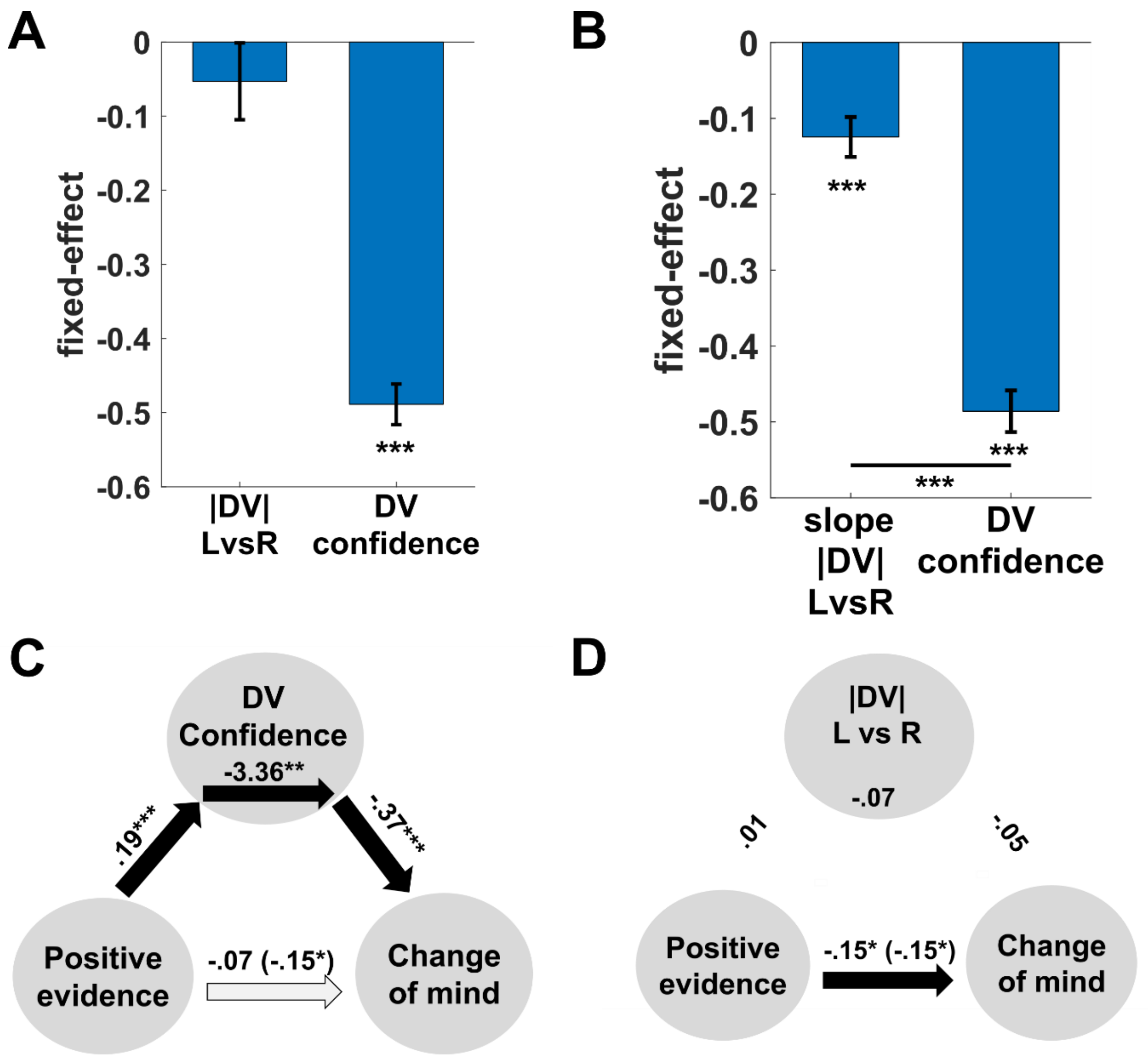

Supplementary Figure 7 *MEG analysis supporting a distinct influence of confidence on changes of mind (n=25 participants).* ***A&B*** *Results of a hierarchical logistic regression predicting changes of mind, based on the strength of neural representation obtained from the decision and confidence classifiers (**A**) and/or slope of the decision classifier (**B**). Fixed effects from hierarchical regression models are presented ± SEM. Hierarchical regression (two-tailed): *** p<.001.* ***C&D*** *Mediation analysis investigating putative neural pathways through which a positive evidence manipulation reduces changes of mind.* ***C*** *Mediation analysis shows that a positive evidence manipulation increases the neural representation of confidence which in turn reduces changes of mind.* ***D*** *In contrast, the effect of positive evidence is not mediated by a change in the neural representation of the decision. DV= decision variable (i.e. distance to classification hyperplane), LvsR= left versus right. Multi-level mediation (two-tailed):*p<.05, ** p<.01, *** p<.001.*

Supplementary Note 7: Predicting post-decision evidence integration from neural representations of pre-decision evidence and confidence

In the main text we focus on the influence of initial confidence (behavioural ratings) on neural metrics of post-decision evidence processing (neural measure), whereas in Supplementary Note 6 we focus on how pre-decisional neural signals of confidence and internal evidence (neural measures) predict later changes of mind (behavioural measure). In a final analysis we sought to directly relate pre-decisional neural signals to neural metrics of the processing of post-decision evidence.

If confidence acts as a top-down controller of the integration of post-decision evidence, neural signatures of initial confidence (measured during the pre-decision phase) should influence post-decision evidence processing by amplifying a confirmation bias (see main text). Indeed, when predicting neural metrics of post-decision evidence processing (slope of the decision classifier) based on pre-decisional neural confidence, initial decision and their interaction, we find a significant influence of an initial decision (β=.05, p=.0009) and a significant interaction between initial decision and neural confidence (β=.04, p=.008) showing that stronger neural signatures of confidence act to boost a confirmation bias. An alternative hypothesis is that the (decisional) evidence people accumulate during the pre-decision phase determines alterations in post-decision evidence processing. When entering the slope obtained from the decision classifier in the pre-decision phase as predictors of post-decision processing, there was again an influence of the initial decision (β=.03, p=.01) but no effect of pre-decision slope or their interaction (p-values>.21). A comparison of these two analyses indicates that neural representations of confidence, rather than decision evidence, are primary modulators of a post-decisional confirmation bias. Together with the behavioural and neural analyses reported in the main text, this result further supports confidence as a critical variable influencing post-decision evidence processing.

Supplementary Figures

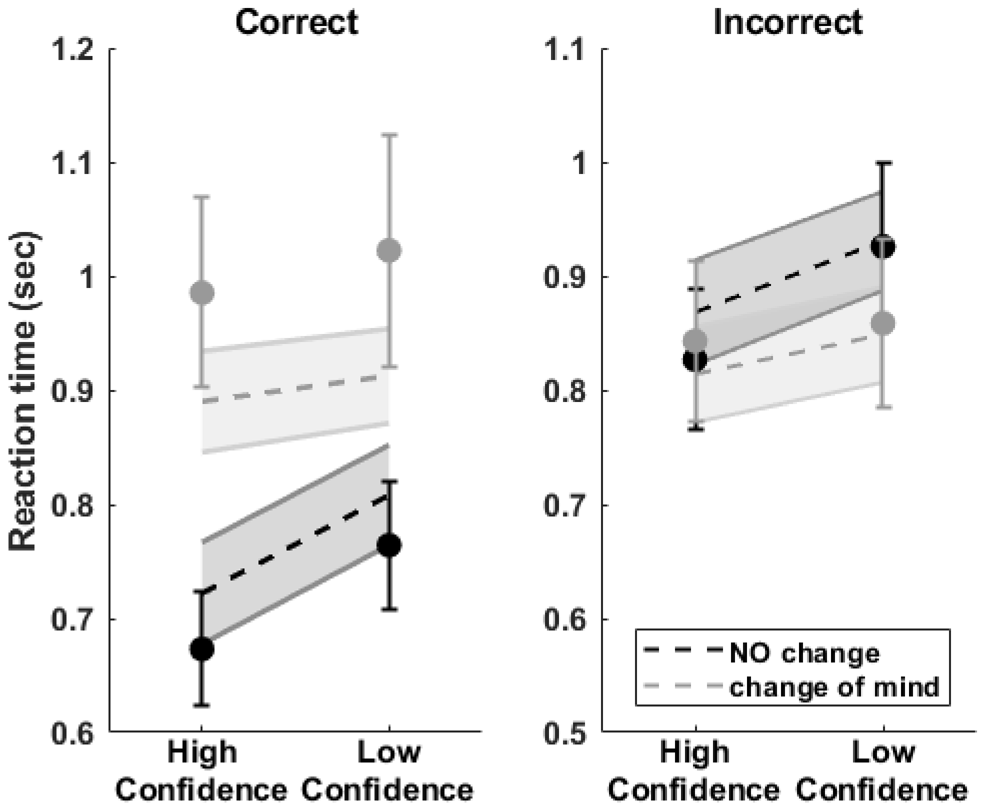

Supplementary Figure 8 *Model simulations (of the best fitting model) reproduce behavioural patterns of reaction times of the second decision* (n= 23 participants). *Data are presented separately for initially correct decisions (left panel) and initially incorrect decisions (right panel), as a function of initial confidence and whether participants changed their mind. Model simulations are shown as dotted lines, behavioral data as solid markers. Data are presented as means ± 95% confidence intervals. Note that the fit appears poor for a subset of conditions containing only small numbers of trials (e.g. for some participants there were only 2 trials with changes of mind after an initially correct decision, with a group median of 12 trials per participant), presumably due to a lack of precision in both measurement and simulation.*

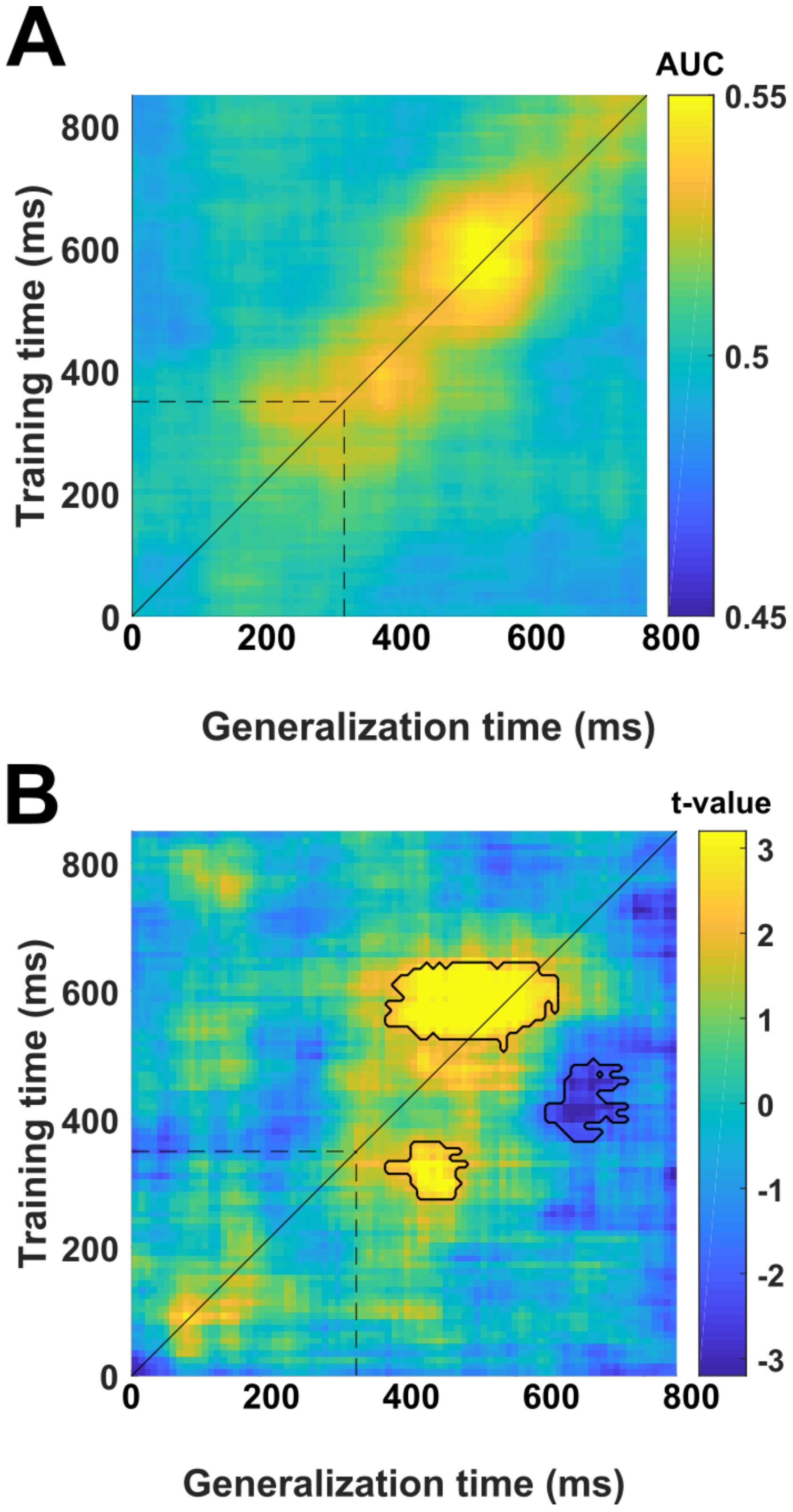

***Supplementary Figure 9 A** Temporal generalization of the decision classifier during the pre-decision phase, i.e. training and testing on all pre-decision time points. Decoding accuracy was obtained as the area under the receiver operating curve (AUC; chance performance=.5). Yellow colours indicate above-chance decoding while blue colours indicate below-chance decoding accuracy. The solid line indicates the major diagonal (i.e. corresponding time*

points in both phases), while the dotted lines indicate the offset of the stimulus. The time window starts with stimulus presentation (0ms) and ends when the response options are presented (850ms). The temporal generalization profile obtained when decoding within the pre-decision phase was similar to that observed when decoding across the pre- and post-decision phases (compare to Supplementary Figure 10A). Highest decodability was observed along the major diagonal, indicating a dynamic representation of the decision, peaking around 550 ms after stimulus onset and declining towards the end of the time window. No statistical tests were conducted on this figure which is provided for illustration purposes. AUC= area under the receiver operating curve. ***B*** *Main effect of confidence on temporal generalization within the pre-decision phase showing a significant modulation (contoured cluster) of confidence on decoding accuracy along the major diagonal, as expected if trials with initially high confidence are also trials in which an upcoming decision is more clearly represented in the pre-decision phase. Notably, the cluster of time points modulated by confidence in the post-decision phase (Figure 4D) showed no such effect during the pre-decision phase, suggesting this post-decisional acceleration cannot be explained by general effects of confidence on evidence accumulation.*

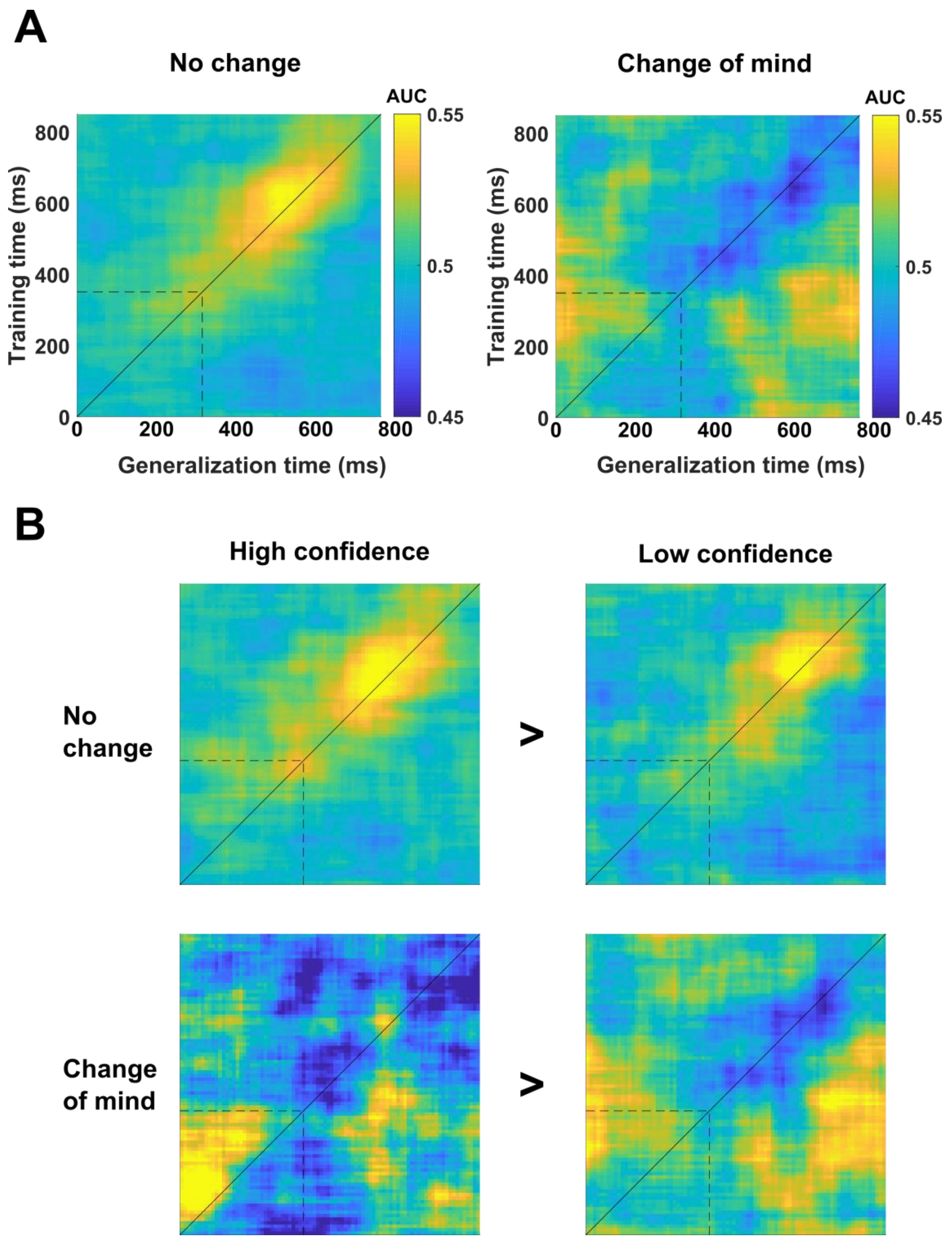

Supplementary Figure 10 *Temporal generalization of decision decoding accuracy across the pre- and post-decision phases. We trained a machine learning classification algorithm to decode participants' initial decisions (left or right) based on pre-decision neural data and tested it on post-decision neural data. Decoding accuracy was based on the area under the receiver operating curve (chance performance=.5) for predicting the initial decision. Yellow colours indicate above-chance decoding while blue colours indicate below-chance decoding accuracy. The solid line indicates the major diagonal (i.e. corresponding time points in both phases), while the dotted lines indicate the offset of the stimulus (pre or post-decision respectively).* ***A*** *Decodability of the initial decision based on whether or not participants went*

on to change their minds (right panel) or not (left panel). As expected, in trials in which subjects did not change their mind, i.e. chose the same option for both the initial and final decision, the same decision continued to be represented in both the pre- and post-decision phases. In contrast, on trials in which people changed their minds, the initially unchosen option began to be represented in the post-decisional phase, leading to below-chance classification accuracy of the initial decision on change of mind trials (i.e. a representation of the opposite decision). The fact that decoding was mainly possible along the major diagonal indicates that similar processing steps are reengaged in both the pre- and post-decision phase. AUC= area under the receiver operating curve. ***B*** *Decodability of the initial decision based on whether or not participants changed their mind, split according to whether the initial decision was held with high or low confidence. The subtraction between the left- and right-hand columns forms the basis of the analysis in Figure 4D in the main text, showing a boost in the representation of choice-consistent evidence when confidence is high.* ***A&B*** *Note that these figures are presented for illustration purposes, and no statistical tests were conducted on each of the individual panels.*

Supplementary Tables

Supplementary Table 1 *Convergence statistics for the model parameters of the winning (full) mode. Values close to 1 indicate successful convergence of the Markov-Chain Monte-Carlo sampler.*

Parameter	Gelman–Rubin statistic
**Bound ~ initial confidence**	1.00
**Drift-rate ~ initial decision**	1.00
**Drift~rate ~ confidence**	1.00
**Drift~rate ~ confidence × initial decision**	1.00
**Drift~rate ~ post-decision evidence strength**	1.00
**Starting point ~ initial decision**	1.00
**Starting point ~ confidence**	1.00
**Starting point ~ confidence × initial decision**	1.00

Supplementary References

1. O'Connell, R. G., Dockree, P. M. & Kelly, S. P. A supramodal accumulation-to-bound signal that determines perceptual decisions in humans. *Nat. Neurosci.* **15,** 1729–1735 (2012).

2. Gold, J. I. & Shadlen, M. N. The neural basis of decision making. *Annu. Rev. Neurosci.* **30,** 535–574 (2007).

3. Kiani, R. & Shadlen, M. N. Representation of confidence associated with a decision by neurons in the parietal cortex. *Science (80-.).* **324,** 759–764 (2009).

4. Braun, A., Urai, A. E. & Donner, T. H. Adaptive History Biases Result from Confidence-weighted Accumulation of Past Choices. *J. Neurosci.* **38,** 2189–17 (2018).

5. Rahnev, D., Koizumi, A., McCurdy, L. Y., D'Esposito, M. & Lau, H. Confidence leak in perceptual decision making. *Psychol. Sci.* **26,** 1664–1680 (2015).

6. Cortese, A., Amano, K., Koizumi, A., Kawato, M. & Lau, H. Multivoxel neurofeedback selectively modulates confidence without changing perceptual performance. *Nat. Commun.* **7,** 1–18 (2016).

7. Peters, M. A. K. *et al.* Perceptual confidence neglects decision-incongruent evidence in the brain. *Nat. Hum. Behav.* **1,** 1–8 (2017).

8. Atiya, N. A. A., Rañó, I., Prasad, G. & Wong-Lin, K. A neural circuit model of decision uncertainty and change-of-mind. *Nat. Commun.* **10,** 2287 (2019).
