# Supplementary material for: Confidence drives a neural confirmation bias
Source: Nat Commun. 2020 May 26;11:2634. doi: 10.1038/s41467-020-16278-6 (PMC7250867; doi:10.1038/s41467-020-16278-6)
Supplement: Supplementary file 3 — Reporting Summary [file 41467_2020_16278_MOESM3_ESM.pdf]

nature research

Corresponding author(s): Max Rollwage

Last updated by author(s): Apr 2, 2020

Reporting Summary

Nature Research wishes to improve the reproducibility of the work that we publish. This form provides structure for consistency and transparency in reporting. For further information on Nature Research policies, see Authors & Referees and the Editorial Policy Checklist .

Statistics

For all statistical analyses, confirm that the following items are present in the figure legend, table legend, main text, or Methods section.

n/a	Confirmed	
☐	☒	The exact sample size (*n*) for each experimental group/condition, given as a discrete number and unit of measurement
☐	☒	A statement on whether measurements were taken from distinct samples or whether the same sample was measured repeatedly
☐	☒	The statistical test(s) used AND whether they are one- or two-sided<br>*Only common tests should be described solely by name; describe more complex techniques in the Methods section.*
☐	☒	A description of all covariates tested
☐	☒	A description of any assumptions or corrections, such as tests of normality and adjustment for multiple comparisons
☐	☒	A full description of the statistical parameters including central tendency (e.g. means) or other basic estimates (e.g. regression coefficient) AND variation (e.g. standard deviation) or associated estimates of uncertainty (e.g. confidence intervals)
☐	☒	For null hypothesis testing, the test statistic (e.g. *F*, *t*, *r*) with confidence intervals, effect sizes, degrees of freedom and *P* value noted<br>*Give P values as exact values whenever suitable.*
☐	☒	For Bayesian analysis, information on the choice of priors and Markov chain Monte Carlo settings
☐	☒	For hierarchical and complex designs, identification of the appropriate level for tests and full reporting of outcomes
☐	☒	Estimates of effect sizes (e.g. Cohen's *d*, Pearson's *r*), indicating how they were calculated

Our web collection on statistics for biologists contains articles on many of the points above.

Software and code

Policy information about availability of computer code

Data collection	The experiment was presented with Matlab r2012a and Psychtoolbox- 3.0.14 using custom code for stimulus presentation. Behavioral data was recorded and saved as .mat files. MEG data was recorded using a 275-channel CTF Omega whole-head gradiometer (VSMMedTech, British Columbia, Canada).
Data analysis	Behavioral data was analyzed with custom code using Matlab r2017b. The Multilevel Mediation and Moderation (M3) Toolbox was used for mediation analysis. Drift-diffusion modeling was conducted in Python 3.4 using the hDDM toolbox (http://ski.clps.brown.edu/hddm_docs/). MEG data was analyzed with custom code in Matlab r2017b, using functions from SPM12 and FieldTrip. To build our support-vector machine classifiers we used the svmtrain/svmpredict routines of libsvm (National Taiwan University, Taiwan; http://www.csie.ntu.edu.tw/~cjlin/libsvm). The custom code for data analysis and computational model fits are available from a dedicated Github repository (https://github.com/MaxRollwage/NatureCommunications).

For manuscripts utilizing custom algorithms or software that are central to the research but not yet described in published literature, software must be made available to editors/reviewers. We strongly encourage code deposition in a community repository (e.g. GitHub). See the Nature Research guidelines for submitting code & software for further information.

Data

Policy information about availability of data

All manuscripts must include a data availability statement. This statement should provide the following information, where applicable:

- Accession codes, unique identifiers, or web links for publicly available datasets
- A list of figures that have associated raw data
- A description of any restrictions on data availability

Fully anonymised data and code for data analysis and computational model fits are available from a dedicated Github repository (https://github.com/MaxRollwage/NatureCommunications).

Field-specific reporting

Please select the one below that is the best fit for your research. If you are not sure, read the appropriate sections before making your selection.

☐ Life sciences ☒ Behavioural & social sciences ☐ Ecological, evolutionary & environmental sciences

For a reference copy of the document with all sections, see nature.com/documents/nr-reporting-summary-flat.pdf

Behavioural & social sciences study design

All studies must disclose on these points even when the disclosure is negative.

Study description	This study investigated the influence of confidence on processing of post-decision evidence and changes of mind. Quantitative data were acquired, including behavioral variables and MEG measures.
Research sample	Each study contained a different group of participants from the subject pool of University College London. We analysed data from 28 participants in study 1 (age: M= 23.8; SD= 6.3; 16 female), 23 participants in study 2 (age: M= 25.7; SD= 7; 12 female) and 25 subjects in study 3 (age: M= 24.6; SD= 4.1; 16 female).<br>The sample was a convenience sample and is therefore not necessarily representative of the general population.
Sampling strategy	A convenience sample was recruited through the subject pool of the University College London. The sample sizes for studies 1-3 were based on comparable published studies of perceptual decision-making, as for instance in:<br><br>Fleming, S. M., van der Putten, E. J. & Daw, N. D. Neural mediators of changes of mind about perceptual decisions. Nat. Neurosci. 1–8 (2018). doi:10.1038/s41593-018-0104-6<br><br>Talluri, B. C., Urai, A. E., Tsetsos, K., Usher, M. & Donner, T. H. Confirmation bias through selective overweighting of choice-consistent evidence. Curr. Biol. 28, 3128–3135 (2018).
Data collection	The experiment was delivered using computer software (Matlab and Psychtoolbox) and all behavioral responses were recorded by the computer. MEG data were also recorded by computer.<br>During the experiments, the participants were alone in the testing room, with the researcher present in an adjacent room.<br>The experiment included a within-subject manipulation so that there was no between-subject manipulation present, thus blinding of the researcher was not necessary.
Timing	Data were acquired between December 2017 and October 2018.<br>Study 1 was collected between December 2017 and February 2018. Study 2 was collected between February 2018 and April 2018. Study 3 was collected between May 2018 and October 2018.
Data exclusions	Participants were excluded based on the following set of pre-defined criteria: using the same initial confidence rating more than 90% of time (N=3 in study 1; N=2 in study 2), performance below 55% or above 87.5% correct decisions in one of the pre-decision evidence conditions indicating non-convergence of the staircase procedure (N=3 in study 1; N=2 in study 2).<br>For MEG study 3, participants conducted an initial behavioural training session before being screened according to the same criteria as in studies 1 and 2. Additionally, data of 4 subjects could not be analysed due to technical problems with recording triggers. As we applied machine learning classification algorithms to the neural data in order to decode decisions (left versus right) and confidence (high versus low) it was important that participants showed relatively balanced responses for these two categories. 2 subjects were excluded because they chose one response more than 80% of the time for either the decision or confidence.
Non-participation	No participants dropped out or declined participation.
Randomization	The experiments focused on within-subjects effects and no randomization into groups was required.

Reporting for specific materials, systems and methods

We require information from authors about some types of materials, experimental systems and methods used in many studies. Here, indicate whether each material, system or method listed is relevant to your study. If you are not sure if a list item applies to your research, read the appropriate section before selecting a response.

Materials & experimental systems

n/a	Involved in the study
[x]	[ ] Antibodies
[x]	[ ] Eukaryotic cell lines
[x]	[ ] Palaeontology
[x]	[ ] Animals and other organisms
[ ]	[x] Human research participants
[x]	[ ] Clinical data

Methods

n/a	Involved in the study
[x]	[ ] ChIP-seq
[x]	[ ] Flow cytometry
[x]	[ ] MRI-based neuroimaging

Human research participants

Policy information about studies involving human research participants

Population characteristics	Please see above.
Recruitment	Participants were recruited through the subject pool of the University College London. The sample was not intended to be representative of the general population. The purpose of the study was not mentioned in the study advertisement.
Ethics oversight	All studies were approved by the Research Ethics Committee of University College London (#1260-003) and all subjects gave written informed consent.

Note that full information on the approval of the study protocol must also be provided in the manuscript.
